# Evolutionary Theories of Aging and Longevity

**DOI:** 10.1100/tsw.2002.96

**Published:** 2002-02-07

**Authors:** Leonid A. Gavrilov, Natalia S. Gavrilova

**Affiliations:** Center on Aging, NORC/University of Chicago, 1155 East 60th Street, Chicago, IL 60637, USA

**Keywords:** evolution, fitness, gerontology, lifespan, longevity, mortality, mutation, reproduction, selection, senescence, survival, trade-offs, antagonistic pleiotropy theory, disposable soma theory, evolutionary theory, life extending mutations, life history theory, mutation accumulation theory, natural selection, programmed death, reproductive cost, reproductive success, single-gene mutations, theories of biological aging

## Abstract

The purpose of this article is to provide students and researchers entering the field of aging studies with an introduction to the evolutionary theories of aging, as well as to orient them in the abundant modern scientific literature on evolutionary gerontology. The following three major evolutionary theories of aging are discussed: 1) the theory of programmed death suggested by August Weismann, 2) the mutation accumulation theory of aging suggested by Peter Medawar, and 3) the antagonistic pleiotropy theory of aging suggested by George Williams. We also discuss a special case of the antagonistic pleiotropy theory, the disposable soma theory developed by Tom Kirkwood and Robin Holliday. The theories are compared with each other as well as with recent experimental findings. At present the most viable evolutionary theories are the mutation accumulation theory and the antagonistic pleiotropy theory; these theories are not mutually exclusive, and they both may become a part of a future unifying theory of aging.

Evolutionary theories of aging are useful because they open new oppor-tunities for further research by suggesting testable predictions, but they have also been harmful in the past when they were used to impose limitations on aging studies. At this time, the evolutionary theories of aging are not ultimate completed theories, but rather a set of ideas that themselves require further elaboration and validation. This theoretical review article is written for a wide readership.

## INTRODUCTION

There is a growing interest in the topic of aging and in the search for a general theory that can explain what aging is and why and how it happens. There is also a need for a general theoretical framework that may allow researchers to handle an enormous amount of diverse observations related to aging phenomena. Empirical observations on aging have become so numerous and abundant that a special encyclopedia, *The Macmillan Encyclopedia of Aging*, is now required for even a partial coverage of the accumulated facts[1]. To transform these numerous and diverse observations into a comprehensive body of knowledge, a general theory of species aging and longevity is required.

A general theory of aging may come in the future from a synthesis between systems theory (reliability theory) and specific biological knowledge. Reliability theory is a general theory about systems failure, which allows researchers to predict age-related failure kinetics for a system of given architecture (reliability structure) and given reliability of its components[2,3]. As for specific biological knowledge, many researchers believe that it could be provided by evolutionary theories of aging based on the Darwinian theory of biological evolution by natural selection[4,5,6,7,8,9,10,11,12,13,14,15,16,17,18,19,20,21,22,23,24,25,26].

The purpose of this article is to provide a balanced scientific discussion of the evolutionary theories of aging, which evolutionary biologists suggest as “the intellectual core of gerontology”[19]. Such a discussion of the evolutionary theories of aging is needed, and it may be particularly useful for students and researchers entering the field of aging studies. This article will also provide them with an orientation to the abundant scientific literature on the evolution of aging, which requires knowledge of the historical background, i.e., how these evolutionary ideas evolved themselves over time. While working on this article, we found that the scientific literature on evolutionary gerontology should be handled with great care because the significance of some publications[27,28] could only be understood in the context of related studies made by other researchers[14,29,30,31,32].

Evolutionary theories of aging and longevity are those theories that try to explain the remarkable differences in observed aging rates and longevity records across different biological species (compare, for example, mice and humans) through interplay between the processes of mutation and selection. The appeal for understanding the biological evolution of aging and lifespan comes also from puzzling observations of the life cycles of some biological species. For example, a bamboo plant reproduces vegetatively (asexually) for about 100 years, forming a dense stand of plants. Then in one season all of the plants flower simultaneously, reproduce sexually, and die. About 100 years later (depending on the exact bamboo species) the process is repeated[33]. This intriguing observation, as well as other similar observations of “suicidal” life cycles of species like pacific salmon[34,35,36], has promoted the idea that sexual reproduction may come with a cost for species longevity. Thus, in addition to mutation and selection, the reproductive cost, or, more generally, the trade-offs between different traits of organisms may also contribute to the evolution of species aging and longevity. The evolutionary theories of aging are closely related to the genetics of aging because biological evolution is possible only for heritable manifestations of aging.

## Why Did It Take So Long?

The logical foundations for modern evolutionary theories of aging were completed only in the 1950s[10,11], almost a century after Darwin suggested his theory of biological evolution[4]. This happened because for many decades the evolution of aging was a puzzling phenomenon to the classic Darwinian theory of evolution by natural selection. Darwin’s theory is based on the idea of random and heritable variation of biological traits between individuals (caused by mutations) with subsequent natural selection for preferential reproduction of those individuals who are particularly fit to a given environment. It is expected (and observed), therefore, that biological evolution acts to increase the fitness and performance of species evolving in successive generations. From this perspective it was difficult to understand why natural selection seemed to result in such bizarrely injurious features as senescence and late-life degenerative diseases instead of eternal youth and immortality. How does it happen that, after having accomplished the miraculous success that led us from a single cell at conception through birth and then to sexual maturity and productive adulthood (including reading this article), the developmental program formed by biological evolution fails even to maintain the accomplishments of its own work?

Another theoretical difficulty in understanding the evolution of aging was the timing problem. Many manifestations of aging happen after the reproductive period of evolving organisms at ages which are beyond the reach of natural selection. It took almost a century to understand that it is exactly this theoretical difficulty of the timing problem that suggests an evolutionary explanation of aging (see below).

The problem of the biological evolution of aging was initially studied in a purely theoretical, nonexperimental way by August Weismann[5,6,7], Ronald Fisher[8], Peter Medawar[9,10], George Williams [11], William Hamilton [12], Brian Charlesworth [13,37], and other researchers. The resulting evolutionary theories of aging were then partially tested by direct evolutionary experiments on laboratory fruit flies[19,22] and on natural populations of guppies[23]. Specifically, the researchers found that aging and lifespan do evolve in subsequent generations of biological species in a theoretically predicted direction depending on particular living conditions. For example, a selection for later reproduction (artificial selection of late-born progeny for further breeding) produced, as expected, longer-lived fruit flies[19] while placing animals in a more dangerous environment with high extrinsic mortality redirected evolution, as predicted, to a shorter lifespan in subsequent generations[22]. Therefore, the early criticism of the evolutionary theory of aging as merely theoretical speculation with limited and indirect supporting evidence obtained from retrospective and descriptive studies has been overturned. On the contrary, the evolutionary plasticity of aging and longevity is now an established experimental fact.

## Evolutionary Theory of Aging vs. Life History Theory

The evolutionary theory of aging may be considered as part of a more general life history theory[20,21], which tries to explain how evolution designs organisms to achieve reproductive success (i.e., avoid extinction). Life history theory is based on mathematical methods of optimization models with specific biological constraints. Among the questions posed and answered by life history theory are[20,21]: Why are organisms small or large? Why do they mature early or late? Why do they have few or many offspring? Why do they have a short or a long life? Why must they grow old and die?

The latter two questions represent the entire scientific agenda of the evolutionary theory of aging. It could be said, therefore, that the evolutionary theory of aging is a subset of the life history theory[32]. On the other hand the evolutionary theory of aging is considered to be the intellectual core of the biodemography of aging and longevity[38].

Current evolutionary explanations of aging and limited longevity of biological species are based on two major evolutionary theories: the mutation accumulation theory[9,10] and the antagonistic pleiotropy theory[11]. These two theories can be summarized as follows:

- Mutation accumulation theory: From the evolutionary perspective, aging is an inevitable result of the declining force of natural selection with age. For example, a mutant gene that kills young children will be strongly selected against (will not be passed to the next generation) while a lethal mutation with effects confined to people over the age of 80 will experience no selection because people with this mutation will have already passed it to their offspring by that age. Over successive generations, late-acting deleterious mutations will accumulate, leading to an increase in mortality rates late in life.
- Antagonistic pleiotropy theory: Late-acting deleterious genes may even be favored by selection and be actively accumulated in populations if they have any beneficial effects early in life.

Note that these two theories of aging are not mutually exclusive, and both evolutionary mechanisms may operate at the same time. The main difference between the two theories is that in the mutation accumulation theory, genes with negative effects at old age accumulate passively from one generation to the next while in the antagonistic pleiotropy theory, these genes are actively kept in the gene pool by selection[32]. The actual relative contribution of each evolutionary mechanism to species aging is not yet determined, and this scientific problem is now the main focus of current research in evolutionary biology.

Interestingly, since the 1950s, no fundamentally new evolutionary theories of aging have been proposed. There were, however, attempts to find a better name for the antagonistic pleiotropy theory and to specify in more detail how one and the same gene could have both deleterious and beneficial effects. In particular, the disposable soma theory was proposed[16,17], which postulated a special class of gene mutations with the following antagonistic pleiotropic effects: these hypothetical mutations save energy for reproduction (positive effect) by partially disabling molecular proofreading and other accuracy promoting devices in somatic cells (negative effect). The authors of the disposable soma theory argued that “it may be selectively advantageous for higher organisms to adopt an energy saving strategy of reduced accuracy in somatic cells to accelerate development and reproduction, but the consequence will be eventual deterioration and death”[17].

While discussing the disposable soma theory, it is important to keep in mind that it was initially proposed to provide evolutionary justification for another theory of aging called Orgel’s error catastrophe theory[39,40]. The error catastrophe theory, which ultimately failed at least in its original form, considered aging a result of a breakdown in the accuracy of protein synthesis within somatic cells[41,42,43,44]. Most researchers agree that the disposable soma theory is a special, more narrowly defined variant of the antagonistic pleiotropy theory of aging[23,32]. This was also admitted by the authors of the disposable soma theory themselves: “The disposable soma theory is, in a sense, a special case of Williams’s (1957) pleiotropic gene hypothesis [antagonistic pleiotropy theory], the gene in question controlling the switch to reduced accuracy in somatic cells. The good effect of the gene is the reduced investment of resources in the soma, while the bad effect is ultimate somatic disintegration, or ageing”[17, p. 540].

After the death of the error catastrophe theory, the disposable soma theory became a widowed concept, adjusting to new realities and ideas. Within the same name, the content of the theory has evolved from specific consideration of the accuracy of protein synthesis in somatic cells to general concerns about “the role of somatic maintenance and repair”[18]. These concerns reiterate an earlier suggestion made by August Weismann in 1889 about “the perishable and vulnerable nature of the soma”[6].

As for other modifications and additional names, the antagonistic pleiotropy theory is also known as the “pay later” theory for reasons that will be discussed later.

## Evolutionary Fundamentalism in Gerontology

In addition to mainstream theoretical and experimental studies of the evolution of aging, there is also a more radical proevolutionary approach with the agenda “to overthrow the present intellectual order of gerontology, and to replace it with one based on evolutionary and genetic foundations”[19].

This ambitious proevolutionary approach considers all other theories of biological aging—such as the free radical theory of aging[45,46], the somatic mutation theory of aging[47,48], the mitochondrial theory of aging[49,50,51], the reliability theory of aging[2,3], and the waste accumulation theory of aging[52]—as far less important to gerontology. Basically, it postulates that “the evolutionary biology of aging, rather than, for example, cell biology, should be the intellectual core of gerontology”[19] “Aging is fundamentally a product of evolutionary forces, not biochemical or cellular quirks.…Aging is a Darwinian phenomenon, not a biochemical one" [53, p. 106].

Apparently, this ambitious proevolutionary doctrine is based on a literal interpretation of the following statement by Theodosius Dobzhansky: "Nothing in biology makes sense except in the light of evolution"[19].

Claims were made[27] that a simple evolutionary model can explain even the observed age-trajectory of mortality curves including the late-life mortality plateaus (tendency of mortality curves to level-off at advanced ages), but other investigators later found these claims to be unsubstantiated [15,29,54,55]. Thus, declarations that the evolutionary theory of aging should have a dominating status among other biological theories of aging remain to be justified. However, the opposite extreme suggestion that late-life mortality deceleration "raises serious questions about the evolution of senescence"[56] has also proved to be unsubstantiated[14].

Scientific ideas on the evolution of aging have themselves evolved over time, and their historical review (see below) is important for a deeper understanding of the subject.

## HISTORICAL EVOLUTION OF SCIENTIFIC IDEAS ON THE EVOLUTION OF AGING

### The Theory of Programmed Death

August Weismann (1834–1914), the great German theorist and experimental biologist of the 19th century, was one of the first biologists to use evolutionary arguments to explain aging. His initial idea was that there exists a specific death-mechanism designed by natural selection to eliminate the old, and therefore worn-out, members of a population[5]. The purpose of this programmed death of the old is to clean up the living space and to free up resources for younger generations. Weismann probably came to this idea while reading the following notes of Alfred Russel Wallace (one of Darwin's contemporaries and a codiscoverer of natural selection), which he later cited in his essay "The Duration of Life"[6]: "…when one or more individuals have provided a sufficient number of successors they themselves, as consumers of nourishment in a constantly increasing degree, are an injury to those successors. Natural selection therefore weeds them out, and in many cases favours such races as die almost immediately after they have left successors."

Weismann enthusiastically accepted and developed further this idea, which also corresponded well with the hiring practices of German universities of that time whereby new candidates had to wait for the death of an old professor in order to obtain positions.

### *Weismann's Evolutionary Theory and the Cell Division Limit*

Suggesting the theory of programmed death, Weismann had to think about the exact biological mechanisms for this death program and came to an idea that there is a specific limitation on the number of divisions that somatic cells might undergo. Specifically, he suggested "that life span is connected with the number of somatic cell generations which follow after each other in the course of an individual life, and that this number, like the life span of individual generations of cells, is already determined in the embryonic cell"[7]. Weismann tried to explain "the different life span of animals by making it dependent on the number of cell generations, which was the norm for each different species"[7].

Remarkably, his purely theoretical speculation over the existence of a cell division limit received a dramatic further development described in more detail elsewhere[2]. In brief, Weismann's theory of the cell division limit was initially challenged by Alexis Carrel[57] and Albert Ebeling[58], who published findings that chicken fibroblast-like cells can be cultivated like microorganisms in cell culture indefinitely (at least for 34 years). Many researchers know now that the concept of cell division limit, which became known as the Hayflick limit, was ultimately confirmed. However, still very few researchers are aware of the true history, described below, of this scientific discovery.

In 1959, H. Earle Swim published an important review article titled “Microbiological aspects of tissue culture” summarizing the results of his earlier experiments on the serial cultivation of 23 strains of fibroblasts derived from normal tissues of the rabbit and chick embryo[59], as well as 51 strains of human fibroblasts derived from foreskin, placenta, testicle, uterus, and embryonic tissues[60]. In this article Swim came to the following fundamentally important conclusions: “…in most instances where growth occurs the cells eventually undergo nonspecific degeneration”[61, p. 145] and “The common experience of many investigators indicates that the early cultivation of cells usually follows a characteristic course which can be conveniently divided into three phases. In phase I the cells proliferate rapidly after an initial lag and usually can be transferred serially without difficulty. Phase II is characterized by a decrease in multiplication to a point where it usually ceases and the cells are eventually lost as a result of nonspecific degeneration”[61, p. 159]. “This was accompanied at first by an increase in the number of granules in the cytoplasm of the cells; later, degenerating cells were observed and their numbers increased progressively until the bottoms of the flasks were covered with a dense layer of cellular debris”[59, p. 201]. The important conclusion was also drawn that when cells stop multiplying, this is not a methodological artifact caused by such factors as inoculum size, toxic media, or their inability to proliferate on glass[60]. Finally, Swim[60] notes, that “infrequently a third stage is recognized by the appearance of actively proliferating cells in phase II cultures”(p. 159). “It should be emphasized that phases I and II represent the usual pattern, while phase III is a relatively rare event”[61, p. 160]. Swim[61] also noted that cells in phase III often differ from the original cells both in morphology and growth pattern. Swim concluded that the unlimited capability of animal cells to divide is an exception rather than the rule, and in many cases the cells which divide without limit little resemble the original normal cells either in morphology or growth pattern[61].

These important findings and conclusions of H. Earle Swim were then successfully elaborated although not extensively cited by Leonard Hayflick (see discussion in [2,62,63,64]). Thus, the Weismann’s idea for the existence of a cell division limit was experimentally confirmed and became known as the Hayflick limit. It has also been suggested that this limit be named the Weismann-Swim-Hayflick (WSH) limit for the reasons discussed earlier[2].

The triumph of Weismann’s idea of a cell division limit did not help, however, to justify his theory of programmed death. The cells stopped dividing, but no programmed cell death occurred[2,65,66]. Moreover, the hypothesis was put forward that this cell growth arrest is in fact beneficial for organism survival because it protects against cancer[65,66]. It was also suggested that the cell division limit may have relevance to another evolutionary theory of aging: the antagonistic pleiotropy theory[67], which will be discussed later.

## *Evolution of Weismann’s Views on the Evolution of Aging*

While discussing Weismann’s evolutionary theory of aging, it is important to know that his scientific views evolved significantly over the course of his life[5,6,7]. When Weismann himself became older, he stopped writing about the “injuriousness” of the old, changed his evolutionary views, and considered old organisms not as harmful but simply neutral for the biological species:

> “…in regulating duration of life, the advantage to the species, and not to the individual, is alone of any importance. This must be obvious to any one who has once thoroughly thought out the process of natural selection. It is of no importance to the species whether the individual lives longer or shorter, but it is of importance that the individual should be enabled to do its work towards the maintenance of the species.…The unlimited existence of individuals would be a luxury without any corresponding advantage”[6].

Remarkably, few scientists noticed that Weismann changed his mind on his own earlier theory[19,68]. Weismann's later, corrected version of the theory—that aging is not an adaptive trait but rather simply a neutral trait—did not receive as much attention as his famous earlier theory of programmed death. After his death in 1914, his name became forever associated with the earlier, not the later, version of his theory. This can be seen in the 1922 edition of the book *The Biology of Death* written by Raymond Pearl:

> "In his famous address of 1881 on the duration of life, Weismann propounded the thesis that death was an adaptation, advantageous to the race, and had arisen and was preserved by natural selection. Probably no more perverse extension of the theory of natural selection than this was ever made" [69, p.43].

## *Testing the Theory of Programmed Death*

Subsequent studies confirmed that Weismann's decision to abandon the initial idea of programmed death was a wise one. Many scientific tests of the programmed death hypothesis were made since Weismann proposed his theory, and some of them are summarized here (for more details[2]).

One way of testing the programmed death hypothesis is based on a comparison of lifespan data for individuals of a single species in natural (wild) and protected (laboratory, domestic, civilized) environments. If the hypothesis is correct, there should not be very large differences in the lifetimes of adult individuals across compared environments. Indeed, for a self-destruction program to arise, take hold, and be maintained in the course of evolution, it must at least have some opportunity, however small, of expression in natural conditions. Consequently, the age at which such a program is "switched on" cannot be too high. Otherwise, because of the high mortality in the wild from predators, hunger, infections, and harsh natural conditions, no one would live to the fateful age, and the self-destruction mechanism could not be expressed. It follows from this that lifespans in even the most favorable conditions cannot significantly exceed the ages reached by the most robust individuals in the wild, if, of course, the tested concept is correct.

Analysis of the actual data reveals, however, a picture completely opposite to what would be expected from the programmed death theory: the lifespans of organisms in protected environments greatly exceed the lifespans observed in natural conditions.

For example, the chaffinch (*Fringilla coelebs*) can live for 29 years in captivity[70]. However, in the wild this is practically impossible, since about half of all birds perish in the course of a year from hunger, cold, disease, and attack by predators, and the mean life span is only 1.4 to 1.5 years[71]. As a result of this high mortality, only 0.1% of the initial number of chaffinches survives to age 11.

Similar observations have been made for field voles (*Microtus arvalis* Pall). In protected laboratory conditions, the average lifespan of voles is about 7 to 8 months while individual specimens survive to 25 months[72]. In the wild, however, the average lifespan of voles is only 1.2 months while only 0.1% of the original number of voles survives to 10 months[72].

As for primates, the median lifespan (age at 50% survival) of chimpanzees (*Pan troglodytes*) living in captivity is between 23 years (males) and 30 years (females), and almost 20% of captive female chimpanzees survive to age 50[73,74]. However, in the wild "natural" conditions the median lifespan is only 8 years, and almost nobody survives to age 50[74].

Observations like these are common for many biological species. Thus, if one attempts to estimate the age of programmed death on the basis of lifespans in laboratory conditions, it becomes clear that no death program could arise or be maintained in evolution if only because it would not be able to come into operation in natural conditions where practically no individual lives to the required age.

The same conclusion is reached from an analysis of data on the human lifespan. At present, the mean life expectancy in developed countries is about 70 to 80 years while the documented record for longevity is 122 years[75,76]. If we take these figures as an estimate for age range in which the death program is switched on, we are forced to admit that such a program could not have arisen in human evolution since, according to palaeodemographic data, virtually nobody survived to such an age. For example, only half of those born in the Late Palaeolithic (30,000 to 10,000 B.C.) reached 8 to 9 years, and only half of those born in the Neolithic (6,000 to 2,000 B.C.) reached 26 years[77]. Moreover, even in the Middle Ages ($9^{th}$ to $12^{th}$ centuries), life expectancy at birth was no greater than 27 to 29 years. Investigations of the skeletons of American Indians have shown that only 4% of the population survived to age 50 even as late as in the $18^{th}$ century[78]. Note for comparison that the probability of surviving to this age in the developed countries is 94 to 96%. If these facts are compared, it is difficult to refrain from posing the following question: Can the guaranteed destruction of a few old people who are chance survivors and doomed in the wild be a sufficient evolutionary basis for the formation and preservation of a special self-destruction program in the human genome? Viewed in this light, the inconsistency of the programmed death hypothesis becomes clear.

The second way to test the programmed death hypothesis is to study the dependence of death rates on an animal’s age. If this theory is correct, then the age-dependence of death rates should change dramatically (explode) after some critical age later in life when the alleged death program comes into action. There should be a breaking point in the dependence of death rates on an animal’s age, and this breaking point should be particularly evident in genetically homogeneous stocks of animals kept in standard laboratory conditions. This prediction was carefully tested by studying hundreds of published life tables compiled for many dozens of different biological species, including humans[2]. This study found that the age-dependence of death rates is very smooth and monotonic without any signs of some critical age or breaking point later in life corresponding to an expected mortality explosion[2]. For example, in humans the death rates start to increase with age as early as age 20, and the mortality trajectory follows a simple monotonic Gompertz-Makeham curve[2]. Moreover, the actual death rates at extreme old ages are even lower than expected according to the monotonic Gompertz-Makeham model[79], which is completely opposite to the prediction of the programmed death theory[2].

Finally, if the question whether death is programmed is approached from the evolutionary point of view, it becomes obvious that special mechanisms for the termination of life could hardly help the individual to fight successfully for his survival and the survival of his progeny. On the contrary, those individuals in whom the action of such a program of self-destruction had been impaired by some spontaneous mutation would quickly displace all the remaining individuals. This is because they would produce more offspring in their longer lifespan, or at least they could increase the survival of their offspring by providing longer parental support.

In 1957 George Williams[11], the author of another evolutionary theory of aging (see discussion later) summarized critical arguments against the programmed death theory (called Weismann's theory for historical reasons). Here is the partial list of his most forceful critical arguments that have now gained even more strength and support:

1. “The extreme rarity, in natural populations, of individuals that would be old enough to die of the postulated death-mechanism”.
2. “The failure of several decades [now a century! L.A.G. & N.S.G.] of gerontological research to uncover any death-mechanism”; (Note that the later discovery of apoptosis, programmed cell death [80,81], is irrelevant to this discussion focusing on the death-mechanism for the whole organism rather than for some of its somatic cells. It is also important to note that apoptosis is mainly at play during early development and thus is not specific to aging.)

3. "The difficulties involved in visualizing how such a feature [program for death] could be produced by natural selection".

There is, however, one good reason why this defunct theory of programmed death should not be ignored or forgotten, and it is that the ghosts of this dead theory are still found everywhere, including in the *Encyclopedia Britannica*, which states that "locked within the code of the genetic material are instructions that specify the age beyond which a species cannot live given even the most favorable conditions"[82, p. 424]. This concept, however, proved to be inconsistent with the observed mortality kinetics at advanced ages (see[2]).

Moreover, attempts are made to revive Weismann's theory of programmed death by suggesting an existence of a special suicide program for the whole organisms (called phenoptosis), which performs "very important functions, purifying…communities of organisms from unwanted individuals"[83]. According to this hypothesis, programmed death "is supposed to prevent the appearance of asocial monsters capable to ruin kin, community and entire population"[83]. Thus, Weismann's evolutionary theory of programmed death still remains an issue of scientific discussions.

As for August Weismann, he should be credited with at least four significant contributions to aging studies:

1. Suggesting the first evolutionary theory of aging that attracted the attention of other researchers.
2. Abandoning his own theory when he understood that it was incorrect. This decision cleaned up the intellectual space for new evolutionary theories of aging.
3. Correctly predicting the existence of a cell division limit without having any data at all (on the basis of theoretical inspiration alone!).
4. Formulating the germ plasm theory, i.e., that the body is strictly divided into two types of cells: the germ cells (sperm or ova cells), which are the only cells transmitting hereditary information to the offspring, as opposed to all other "somatic" cells, with a prophetic claim of "the perishable and vulnerable nature of the soma"[6]. The relation of this idea to the more recent disposable soma theory of aging[16,17] is obvious.

## Mutation Accumulation Theory of Aging

This evolutionary theory, suggested by Peter Medawar[9,10], considers aging as a byproduct of natural selection. According to this theory, aging is a nonadaptive trait because natural selection is negligent of events that occur in a few long-lived animals that provide little additional contribution to offspring numbers. This explanation of aging is similar to the evolutionary explanation of vision deterioration and ultimate blindness of cave animals: if some function cannot be used to provide reproductive advantage, it will not be supported by selection pressure and maintained in future generations.

The probability of an individual reproducing depends on age. It is zero at birth and reaches a peak in young adults. Then it decreases due to the increased probability of death linked to various external (predators, illnesses, accidents) and internal (senescence) causes. In such conditions, deleterious mutations expressed at a young age are severely selected against due to their high negative impact on fitness (number of offspring produced). On the other hand, deleterious mutations expressed only later in life are relatively neutral to selection because their bearers have already transmitted their genes to the next generation. Note that mutations can affect fitness either directly or indirectly. For example, a mutation increasing the risk for leg fracture, due to a low fixation of calcium, may be indirectly as deleterious to fitness as a mutation directly impairing the eggs nesting in the uterus[32]. From an evolutionary perspective, it does not really matter if the organism is at risk not to reproduce because many spontaneous abortions occur, or because it becomes an easy prey for a predator (in nature) or a criminal (in society).

According to this theory, persons loaded with a deleterious mutation have fewer chances to reproduce if the deleterious effect of this mutation is expressed earlier in life. For example, patients with progeria (a genetic disease with symptoms of premature aging) live for only about 12 years[84], and, therefore, cannot pass their mutant genes to subsequent generations. In such conditions, the progeria stems only from new mutations and not from the genes of parents. By contrast, people expressing a mutation at older ages can reproduce before the illness occurs, such as the case with familial Alzheimer's disease. As an outcome, progeria is less frequent than late diseases such as Alzheimer's disease because the mutant genes responsible for the Alzheimer's disease are not removed from the gene pool as readily as progeria genes and can thus accumulate in successive generations[32]. In other words, the mutation accumulation theory predicts that the frequency of genetic diseases should increase at older ages.

Mutation accumulation theory allows researchers to make several nontrivial testable predictions. In particular, this theory predicts that the dependence of progeny lifespan on parental lifespan should not be linear, as is observed for almost any other quantitative trait demonstrating familial resemblance (for example, body height). Instead, this dependence should have an unusual nonlinear shape with an increasing slope for the dependence of progeny lifespan on parental lifespan for longer-lived parents. This prediction follows directly from the key statement of this theory that the equilibrium gene frequency for deleterious mutations should increase with age at onset of mutation action because of weaker (postponed) selection against later-acting mutations[13]. The term “equilibrium gene frequency” refers here to the ultimate, time-independent gene frequency, which is determined by mutation-selection balance (equilibrium between mutation and selection rates).

According to the mutation accumulation theory, one would expect the genetic variability for lifespan (in particular, the additive genetic variance responsible for familial resemblance) to increase with age[13]. The additive genetic variance refers here to variance of additive genetic origin (variation due to the additive effects of genes on a particular trait in a genetically heterogeneous population), and this variance increases with an increase in mutation frequencies[13]. The predicted increase in the additive genetic variance could be detected by studying the ratio of additive genetic variance to observed phenotypic variance. This ratio (the so-called narrow-sense heritability of lifespan) can be easily estimated as the doubled slope of the regression line for the dependence of offspring lifespan on parental lifespan[85,86]. Thus, if the ages at death were indeed determined by accumulated late-acting deleterious mutations, one would expect this slope to become steeper with higher parental ages at death[87]. This prediction was tested through the analysis of genealogical data on familial longevity in European royal and noble families, data well known for their reliability and accuracy. It was found that the regression slope for the dependence of offspring lifespan on parental lifespan increases with parental lifespan, exactly as predicted by the mutation accumulation theory[87,88,89].

The current status of the mutation accumulation theory could be characterized as a productive working hypothesis pending further validation.

## “Pay Later” Theory: Antagonistic Pleiotropy Theory of Aging

The theory of antagonistic pleiotropy is based on two assumptions. First, it is assumed that a particular gene may have an effect not on one trait only but on several traits of an organism (pleiotropy). The second assumption is that these pleiotropic effects may affect individual fitness in opposite (antagonistic) ways. This antagonistic pleiotropy theory was proposed by George Williams[11], who noticed that “natural selection may be said to be biased in favor of youth over old age whenever a conflict of interests arises.”

According to Williams, this conflict arises from “pleiotropic genes that have opposite effects on fitnesses at different ages.…Selection of a gene that confers an advantage at one age and a disadvantage at another will depend not only on the magnitudes of the effects themselves, but also on the times of the effects. An advantage during the period of maximum reproductive probability would increase the total reproductive probability more than a proportionately similar disadvantage later on would decrease it. So natural selection will frequently maximize vigor in youth at the expense of vigor later on and thereby produce a declining vigor (aging) during adult life”[11]. These initially verbal arguments of George Williams were later proved mathematically by Brian Charlesworth[13].

In other words, Williams suggested the existence of so-called pleiotropic genes (demonstrating favorable effects on fitness at young ages and deleterious ones at old age) that could explain the aging process. Such genes will be maintained in the population due to their positive effect on reproduction at young ages despite their negative effects at old postreproductive age (their negative effects in later life will look exactly like the aging process).

For the purpose of illustration, suppose that there is a gene increasing the fixation of calcium in bones. Such a gene may have positive effects early in life because the risk of bone fracture and subsequent death is decreased, but such a gene may have negative effects later in life because of increased risk of osteoarthritis due to excessive calcification[32]. In the wild, such a gene has no actual negative effect because most animals die long before its negative effects can be observed. There is then a trade-off between an actual positive effect at a young age and a potential negative one at old age; this negative effect may become effective only if animals live in protected environments such as zoos or laboratories[32].

Another example of antagonistic pleiotropy refers to replicative cellular senescence (cell division limit), which is known to suppress tumorigenesis by switching cells into a state of arrested growth[65,66,67]. This very process that suppresses tumorigenesis early in life, however, may promote cancer in later life because senescent cells stimulate other premalignant and malignant cells to proliferate and to form tumors[67]. Here again there is a trade-off between the earlier protective effect of growth arrest because of cellular senescence and the later detrimental effect caused by cancer promotion.

The antagonistic pleiotropy theory explains why reproduction may come with a cost for species longevity and may even induce death (see the story on bamboo plants and “suicidal” salmon life cycles at the beginning of this article). Indeed, any mutations favoring more intensive reproduction (more offspring produced) will be propagated in future generations even if these mutations have some deleterious effects in later life. For example, mutations causing overproduction of sex hormones may increase the sex drive, libido, reproductive efforts, and success, and therefore they may be favored by selection despite causing prostate cancer (in males) and ovarian cancer (in females) later in life. Thus, the idea of reproductive cost, or more generally of trade-offs, between different traits of the organism follows directly from antagonistic pleiotropy theory.

The trade-offs between reproduction (reproductive success, fitness, vigor) and longevity were predicted by George Williams as “testable deductions from the theory”[11]. Specifically, he predicted: “rapid individual development should be correlated with rapid senescence. Reproductive maturation is the most important landmark in the life-cycle for the evolution of senescence. Senescence may theoretically begin right after this stage in development. So the sooner this point is reached, the sooner senescence should begin, and the sooner it should have demonstrable effects”[11]. This prediction was tested in the fruitfly *Drosophila melanogaster* and, contrary to this prediction, no linear relationship was found between the speed of development and longevity[90].

Another prediction of the trade-offs between reproductive capacity (vigor) and longevity was made by George Williams in the following way: “Successful selection for increased longevity should result in decreased vigor in youth”[11].

These predictions were tested later in selection experiments using *D. melanogaster*. By postponing reproduction to later ages, the intensity of selection on the later stages of life was increased. This selection for late reproduction increased lifespan of the selected populations as Williams predicted[19,91]. Note, however, that in this experimental design the unintended direct selection for longevity also occurs because late reproducing individuals need to be alive at the time of selection. Therefore, these findings of increased longevity are inconclusive because these experiments fail to separate the effects of selection for late reproduction on longevity from direct effects of selection for increased lifespan[92]. Interestingly, the increase in longevity was accompanied by an evolutionary decline in fertility early in adult life, confirming the prediction of the antagonistic pleiotropy theory[19,22,91]. A trade-off between fecundity and longevity was also observed when fruitflies were selected directly for longevity[25,92]. In another selection experiment with different levels of extrinsic mortality, the descendants from populations with low extrinsic mortality demonstrated increased longevity, longer development times, and decreased early fecundity[22]. The general finding from these selection experiments in fruitflies is therefore that increased longevity is associated with depression of fitness in early life, as Williams predicted.

The search for the trade-offs between longevity and reproduction was made also in experiments with soil-dwelling round worms (the nematode *Caenorhabditis elegans)* where a number of long-lived mutants have been identified. When long-lived mutants were reared together with normal (wild-type) individuals under standard culture conditions, neither of them exhibited a competitive advantage, contrary to theoretical evolutionary predictions[93,94]. Only when cultures were exposed to starvation cycles (alternatively fed and starved, mimicking field conditions in nature) did the wild-type worms outcompete (outnumber) the long-lived mutant[94]. These findings demonstrate that increasing lifespan may exhibit some fitness cost only in harsh conditions (cycles of severe starvation), thereby providing limited support for the antagonistic pleiotropy theory of aging.

Data on humans proved to be even less convincing. One study found that long-lived people (women in particular) did have impaired fertility, as predicted by antagonistic pleiotropy theory in general and by disposable soma theory in particular[28]. However, serious methodological flaws were found later in that study[30,31], and its findings proved to be inconsistent with findings of many other researchers including historical demographers analyzing human data (see reviews[30,32]).

When claims are made that many long-lived women are childless[28], it is important to cross-check the data and to make sure that the lack of children is real rather than caused by data incompleteness. For example, we examined the 335 claims of childless women in the dataset used by Westendorp and Kirkwood[28]. When we cross-checked these claims with other professional sources of data described elsewhere[31,89], we immediately found that at least 107 allegedly childless women (32%) did have children! Here are a few illustrative examples for our findings:

- Henrietta Kerr (1653–1741) was apparently childless in the dataset used by Westendorp and Kirkwood[28] and lived 88 years. Our cross-checking revealed that she did have at least one child, Sir William Scott (2nd Baronet of Thirlstane, died on October 8, 1725).
- Charlotte Primrose (1776–1864) was also considered childless in the initial dataset and lived 88 years. Our cross-checking of the data revealed that in fact she had as many as five children: Charlotte (1803–1886), Henry (1806–1889), Charles (1807–1882), Arabella (1809–1884), and William (1815–1881).
- Wilhelmina Louise von Anhalt-Bernburg (1799–1882), apparently childless, lived 83 years. In reality, however, she had at least two children, Alexander (1820–1896) and Georg (1826–1902).

These illustrative examples demonstrate that extreme caution should be exercised when claims for common childlessness among long-lived women are made. The incompleteness of genealogies can generate a spurious increase in the prevalence of claimed childless women among those who live long lives. This happens because children are often not mentioned in particularly obscure, side branches of genealogical trees (remote relatives). It is also known that long-lived people have more chances of being mentioned in incomplete genealogies because of the longer paper trail in various archives generated during their long life. Thus, incompleteness of genealogies generates two types of biases producing a spurious rise in claimed childlessness with increased lifespan: underreporting of children and inflated prevalence of long-lived people.

We sorted the dataset used by Westendorp and Kirkwood[28] in order to determine exactly who are these "childless" long-lived women. We found that most of these claims belong to obscure, poorly studied branches of genealogical trees rather than to the well-documented families (kings, princes, counts, and earls). The percentage of "childlessness" among long-lived women (lifespan above 80 years, born before 1800) dropped from 43% in poorly documented families to 23% in better-studied families (where husbands belonged to nobility ranks higher than barons and baronets). These findings indicate that data quality concerns should be taken seriously when studying evolutionary trade-offs between fertility and longevity in human populations.

It is also important to understand that childlessness cannot be an indicator of poor biological fecundability unless other social explanations are ruled out. For example, woman can be perfectly fertile at younger ages but still childless simply because she married too late. This is illustrated by the following case taken from the dataset analyzed by Westendorp and Kirkwood [28]:

- Anderlechtia Clarissa Chetwynd (1765–1855), allegedly childless, lived 90 years. Our cross-checking did not challenge her childless status but revealed a trivial explanation for her infertility: she married at age 41 years (May 2, 1806) and therefore could hardly have any children.

We found at least 17 other cases in the same dataset when childlessness could be explained by the late age of women at their marriage (first marriage after 37 years).

Furthermore, a perfectly fertile woman can nevertheless be childless if she married an old infertile man. Here is an example from the same dataset:

- Mary Woolley Gibbings (1799–1889), allegedly childless, lived 90 years. Our cross-checking did not challenge her childlessness but revealed a simple explanation: her husband, Sir Cotton Stapleton (1773–1865), 6$^{th}$ Baronet, was 65 years old when they married on October 2, 1838. It would not be fair to blame this long-lived lady for her infertility!

We found at least 19 similar cases in the same dataset when women's childlessness was probably caused by her husband's infertility (husband's age at marriage was more than 50 years).

A fertile woman can also be childless if her husband died shortly after the marriage. This is illustrated by the following example:

- Catherine von Oldenburg (1538–1620), allegedly childless, lived 82 years. Our data cross-checking did not challenge her childlessness but revealed a possible trivial explanation: her husband, Albert II von Hoya (1526–1562/63) died shortly after they married on June 1, 1561.

We found at least six additional cases in the same dataset when childlessness could be caused by early death of the husband living less than 3 years after the marriage.

Based on these examples, we can make the following general conclusion: the “discovery” of the evolutionary trade-offs between human biological fecundability and human longevity[28] may be an artifact caused by data incompleteness and by ignoring other, nonbiological explanations[30,31]. Therefore, more additional studies on this subject are required.

## IMPLICATIONS FOR AGING RESEARCH

Evolutionary biologists were always very generous with gerontologists in providing advice and guidance on how to do aging research “in directions that are likely to be fruitful”[11]. Surprisingly, this generous intellectual assistance proved to be extremely injurious for aging studies in the past. This happened because evolutionary theory was interpreted in such a way that the search for single-gene mutations (or life-extending interventions) with very large positive effects on lifespan was considered a completely futile task, destined for failure for fundamental evolutionary reasons. Researchers were convinced by the forceful evolutionary arguments of George Williams that “…natural selection will always be in greatest opposition to the decline of the most senescence-prone system” and, therefore:

> “Senescence should always be a generalized deterioration, and never due largely to changes in a single system.…This conclusion banishes the ‘fountain of youth’ to the limbo of scientific impossibilities where other human aspirations, like the perpetual motion machine and Laplace's ‘superman’ have already been placed by other theoretical considerations. Such conclusions are always disappointing, but they have the desirable consequence of channeling research in directions that are likely to be fruitful”[11].

As a result of this triumphant evolutionary indoctrination, many exciting research opportunities for lifespan extension were squandered for half a century until the recent and astonishing discovery of single-gene mutants with profoundly extended longevity was ultimately made [95,96,97,98,99,100,101], despite all discouraging predictions and warnings based on evolutionary arguments.

Recent discoveries of lifespan-extending mutations are spectacular. A single-gene mutation (*daf-2*) more than doubles the lifespan of nematodes, keeping them active, fully fertile (contrary to predictions of the disposable soma theory), and having normal metabolic rates[99]. Another single-gene mutation, called *methuselah,* extends the average lifespan of fruitflies by about 35%, enhancing also their resistance to various forms of stress, including starvation, high temperature, and toxic chemicals[100]. Finally, a single-gene mutation was found in mice extending their lifespans by about 30% and also increasing their resistance to toxic chemicals[101]. Transgenic methods for increasing lifespan are also developed[102]. Researchers involved in these studies came to the following conclusion:

> “The field of ageing research has been completely transformed in the past decade.…When single genes are changed, animals that should be old stay young. In humans, these mutants would be analogous to a ninety year old who looks and feels forty-five. On this basis we begin to think of ageing as a disease that can be cured, or at least postponed.…The field of ageing is beginning to explode, because so many are so excited about the prospect of searching for—and finding—the causes of ageing, and maybe even the fountain of youth itself”[103].

Now, when the single-gene life-extending mutations have been found, evolutionary biologists are presented with the task of reconciling these new discoveries with the evolutionary theory of aging, and no doubt they will ultimately succeed.

However, gerontologists will also have to learn a lesson from the damage caused by decades of misguided research, when the search for major life-extending mutations and other life-extension interventions was equated by evolutionary biologists to a construction of perpetual motion machine. Perhaps some wisdom from this lesson can be found in the title of a recent scientific review on the evolution of aging[104]: “Evolutionary theories of aging: handle with care.” Evolutionary theories of aging are useful when they open new opportunities for research by suggesting testable predictions, but they should never be used to impose limitations on aging studies. This is because the evolutionary “theories” of aging are not in fact completed theories, but rather a set of ideas that themselves require further elaboration and validation.

## ACKNOWLEDGMENTS

This work was made possible thanks to the support from National Institute on Aging grants. We would also like to thank Brian Whiteley for useful editorial suggestions and two anonymous reviewers for constructive criticism.
